# Author response to Zupan et al., Letter to the editor regarding “Intracranial hemorrhage after ischemic stroke in patients on direct oral anticoagulants: results from a prospective observational study” by Purrucker et al

**DOI:** 10.1186/s42466-026-00483-7

**Published:** 2026-06-02

**Authors:** Jan Purrucker, Kirsten Haas, Marilen Sieber, Viktoria Rücker, Peter U. Heuschmann, Roland Veltkamp

**Affiliations:** 1 Department of Neurology, Heidelberg University Hospital, Heidelberg, Germany; 2 Institute of Clinical Epidemiology and Biometry, Julius-Maximilians-Universität Würzburg (JMU), Würzburg, Germany; 3 Clinical Trial Center Würzburg, University Hospital Würzburg, Würzburg, Germany; 4 Institute for Medical Data Science, University Hospital Würzburg, Würzburg, Germany; 5 Department of Neurology, Alfried-Krupp Hospital, Essen, Germany; 6 Department of Brain Sciences, Department of Brain Research, Imperial College London, Room10L17, 10th Floor Lab Block, Charing Cross Campus, Margravine Road, London, W6 8RP UK

Dear Editor,

We thank Zupan and colleagues for their insightful comments on our RASUNOA-Prime study [1]. We share their view that the management of acute ischemic stroke in patients receiving oral anticoagulation is among the most pressing unresolved questions in contemporary stroke care. Evidence in this field has so far relied mainly on retrospective data. RASUNOA-Prime, with its study used a prospective, multicenter design, pre-specified recruitment strategy enrolling anticoagulated and non-anticoagulated patients in parallel, and a centralized blinded imaging assessment adds further support to a more individualized assessment of eligibility for intravenous thrombolysis in anticoagulated patients. Nevertheless, our findings should be interpreted with appropriate caution. While the low rate of symptomatic intracranial hemorrhage in anticoagulated patients in RASUNOA-Prime is reassuring, our observational study was not designed to provide a definitive answer regarding the safety of intravenous thrombolysis in all anticoagulated patients, particularly not in the high-risk scenarios encountered in emergency practice.

We also agree that the lower stroke severity observed in patients receiving DOACs is clinically plausible. However, given the observational nature of our study, this finding should not be overinterpreted mechanistically, as residual confounding and treatment selection may have contributed.

Importantly, we share the authors’ view that the available evidence increasingly supports a more nuanced assessment of recent DOAC use than current rigid exclusion rules imply. In our opinion, the key implication of our study is not that established contraindications should be discarded altogether, but that decision-making in this setting should move beyond automatic assumptions toward individualized evaluation based on clinical presentation, imaging, and—where available—laboratory information.

The usefulness of reversal of anticoagulation before thrombolysis (e.g. idarucizumab in patients treated with dabigatran) [2] remains an unresolved issue [3]. Our study was not designed to determine the incremental value of reversal treatment, but the asymmetry between dabigatran-specific reversal and the lack of an equivalent routine strategy for factor Xa inhibitors highlights the need for better prospective evidence and more harmonized recommendations.

In summary, we agree with Zupan and colleagues that the field is moving toward a more differentiated approach to anticoagulated patients with acute ischemic stroke. Our data support this development, while also underscoring that prospective comparative studies, ideally including randomized controlled trials, remain essential before practice recommendations can be changed with confidence.

## Data Availability

Not applicable.
